# Ferroptosis: Mechanisms, Comparison with Cuproptosis and Emerging Horizons in Therapeutics

**DOI:** 10.32604/or.2025.069049

**Published:** 2025-12-30

**Authors:** Shujie Yin, Zong Li, Wen-Bin Ou

**Affiliations:** 1 Department of Biopharmaceutics, Zhejiang Provincial Key Laboratory of Silkworm Bioreactor and Biomedicine, Zhejiang Sci-Tech University, Hangzhou, 310018, China; 2 Zhejiang Provincial Engineering Research Center of New Technologies and Applications for Targeted Therapy of Major Diseases, College of Life Sciences and Medicine, Zhejiang Sci-Tech University, Hangzhou, 310018, China

**Keywords:** Ferroptosis, cuproptosis, therapy, cancers

## Abstract

Ferroptosis is an iron-dependent, excessive lipid peroxidation-driven form of regulated cell death. The core mechanisms of ferroptosis include lipid peroxidation cascade, System X_c_^−^-glutathioneglutathione peroxidase 4 axis, iron and lipid metabolism chaos, the NAD(P)Hferroptosis suppressor protein 1—ubiquinone axis, and GTP cyclohydrolase 1 tetrahydrobiopterin-dihydrofolate reductase axis. Cuproptosis is triggered by copper ions and involves ferredoxin 1-mediated aggregation of lipoylated proteins, differing fundamentally from ferroptosis. Both ferroptosis and cuproptosis exhibit dual roles (promote or inhibit) in cancers. And the sensitivity of different cancer types to ferroptosis varies, which may depend on special metabolic signatures (e.g., E-cadherin loss causes epithelial–mesenchymal transition, making tumors gain resistance to ferroptosis) and expression of antioxidant defense regulators (e.g., high expression of Acyl-CoA synthetase long-chain family member 4 and lncFASA make tumors easily sensitive). At present, traditional Chinese herbal medicine, combination therapy, and nano-delivery technology correlated with ferroptosis are being hotly studied by researchers in order to realize clinical translation of ferroptosis. In this review, we have summarized the core mechanisms of ferroptosis, ferroptosis differences from cuproptosis, its impact on cancers, and its translational implications in cancer therapy, helping readers quickly get the new information and horizons on them.

## 1 Introduction

Cell death can be divided into accidental cell death (ACD) and regulated cell death (RCD) according to morphological, biochemical, and functional differences [1]. ACD happens instantaneously under extreme conditions, whereas RCD is regulated by a particular molecular mechanism regardless of extracellular perturbations. Hence, RCD plays an important role in organic homeostasis and development throughout our whole life and has practical application in the treatment of diseases such as cancer. Formerly, RCD covered ferroptosis, cuproptosis, apoptosis, necroptosis, pyroptosis, parthanatos, entosis, NETosis and so on [1,2].

Ferroptosis, a recently identified form of RCD, shows unique features [3]. During ferroptosis, cells display characteristic mitochondrial alterations, including shrinkage, increased electron density, cristae disappearance, and outer membrane rupture, distinguishing it from other death modalities [3]. The process is induced by intracellular microenvironment variation, chiefly associated with toxic lipid peroxide accumulation resulting from reactive oxygen species (ROS) overproduction and iron metabolism dysregulation [4]. Although both ferroptosis and cuproptosis depend on metal ions, their characteristics and underlying mechanisms are distinct. Cuproptosis is a new term and concept proposed in 2022, which is induced by copper-driven lipoylated protein aggregation [5]. Currently, promising cancer drugs based on ferroptosis and cuproptosis are constantly emerging. Diverse therapy (e.g., integrating with nanotechnology) and novel direction (e.g., bioactive compounds from Chinese herbs) bring more solutions to the issues of drug resistance and serious adverse events in existing medications. This review comprehensively outlines the core mechanisms of ferroptosis, its differences from cuproptosis, and its recent translational implications in cancer therapy.

## 2 Core Mechanisms in Ferroptosis

**Figure 1:**
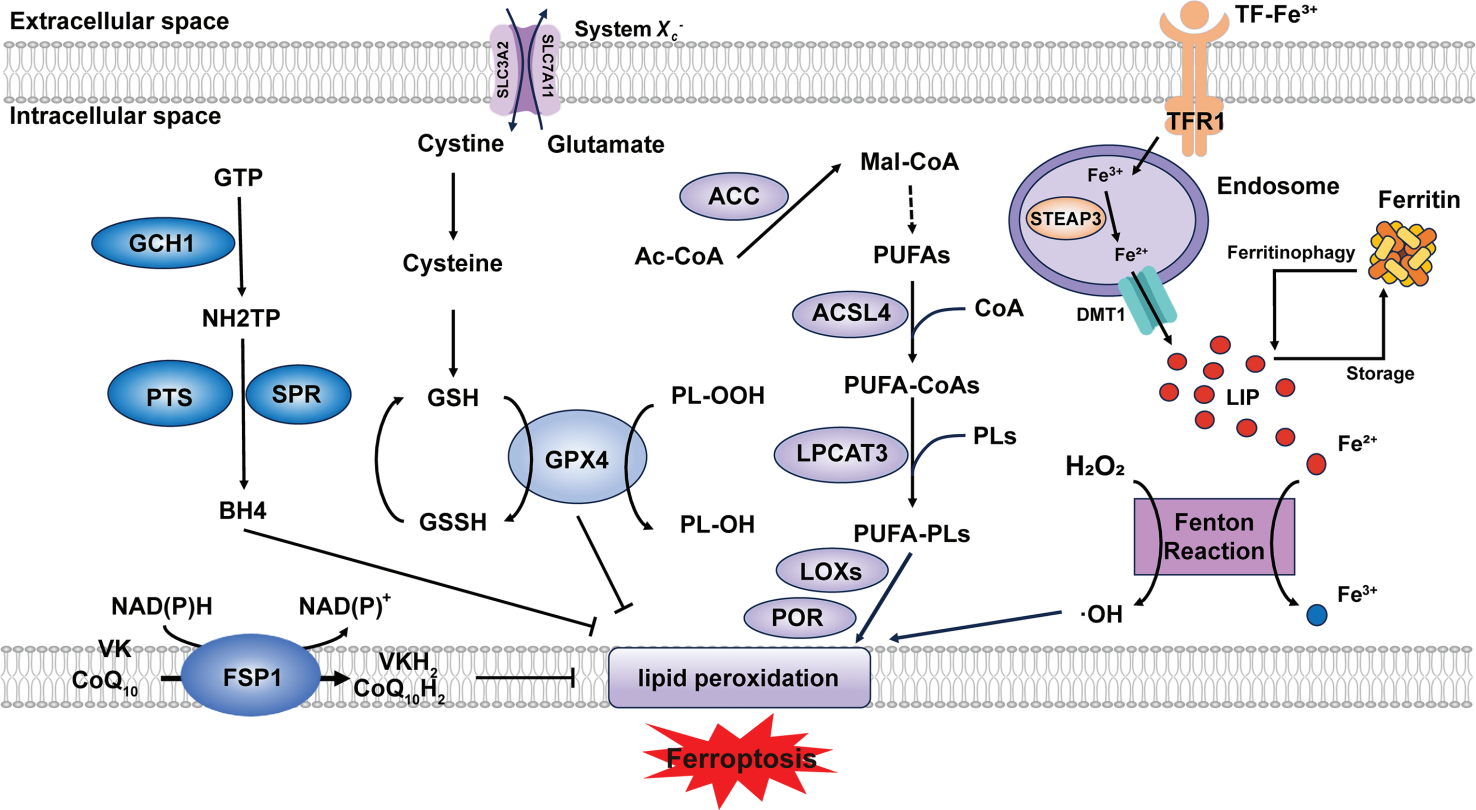
Core mechanisms of ferroptosis. Lipid peroxidation is driven by PUFA-PLs synthesis and excessive iron toxicity. The defense systems of ferroptosis mainly include System X_c_^−^-GSH-GPX4 axis, NAD(P)H-FSP1-CoQ_10_ axis and GCH1-BH4-DHFR axis. When the activity of lipid peroxidation surpasses the detoxification capabilities provided by the defense systems, ferroptosis occurs. Abbreviation: GTP, guanosine triphosphate; GSSG, oxidized glutathione; Ac-CoA, acetyl-coenzyme A; Mal-CoA, malonyl-coenzyme A. Some of the materials in the picture are taken from the drawing software “Biorender”

To date, the molecular mechanisms underlying ferroptosis have been progressively elucidated (Fig. 1). Beyond the well-established core machinery of ferroptosis, newly identified upstream regulators and cellular defense mechanisms are continuously expanding the ferroptosis regulatory network. Here, we briefly outline its core mechanisms and new ferroptosis-related relationships.

### 2.1 The Lipid Peroxidation Cascade

Acyl-CoA synthetase long-chain family member 4 (ACSL4) and lysophosphatidylcholine acyltransferase 3 (LPCAT3) are two key enzymes in the biosynthesis of polyunsaturated fatty acid-containing phospholipids (PUFA-PLs). ACSL4 catalyzes the conjugation of polyunsaturated fatty acids (PUFAs) with coenzyme A (CoA) to form PUFA-CoAs. Subsequently, LPCAT3 mediates the re-esterification of these PUFA-CoAs into phospholipids (PLs), generating PUFA-PLs [6]. Although PUFA-PLs contribute to membrane fluidity, their bis-allylic moieties render them susceptible to oxidation [7], which can be regarded as the foundation of lipid peroxidation. Current evidence indicates that the oxidation process primarily depends on two major pathways: (1) Free iron–induced fenton reaction (Fe^2+^ + H_2_O_2_ → Fe^3+^ + ·OH + OH^−^) produces excessive ROS [8]. (2) Enzyme-mediated reaction. Lipoxygenases (LOXs) and cytochrome P450 oxidoreductase (POR) are capable of triggering lipid peroxidation through their catalytic dioxygenation of PUFAs by molecular oxygen [9]. Consequently, phospholipid hydroperoxides (PLOOHs) are produced, leading to the rupture of organelles and cell membranes.

### 2.2 Dynamic Defense Systems

#### 2.2.1 Canonical Pathways

System X_c_^−^, a heterodimeric antiporter composed of solute carrier family 7 member 11 (SLC7A11) and solute carrier family 3 member 2 (SLC3A2) subunits, functions as a cystine/glutamate exchanger and plays a pivotal role in cellular antioxidant defense [10]. System X_c_^−^ transports cystine into the cell against glutamate at 1:1 stoichiometry [11]. Then cystine is reduced to cysteine to serve as a critical precursor for glutathione biosynthesis [12]. Glutathione peroxidase 4 (GPX4) utilizes reduced glutathione (GSH) as an essential cofactor to catalyze the reduction of cytotoxic lipid hydroperoxides to their corresponding non-toxic lipid alcohols, thereby preventing lethal lipid peroxidation in cells [13]. Consequently, pharmacological or genetic inhibition of either System X_c_^−^ or GPX4 represents approaches to induce ferroptosis. Ubiquitin-specific protease 8 (USP8) represents a novel regulatory mechanism of ferroptosis by modulating GPX4 stability, whose inhibition destabilizes GPX4 and expression stabilizes GPX4 [14]. Zinc finger DHHC-domain containing protein 8 (zDHHC8) palmitoylates GPX4 at Cys75, whose degradation attenuates GPX4 palmitoylation and enhancing ferroptosis sensitivity [15]. The long non-coding RNA (lncRNA) LncFASA binds to the Ahpc-TSA domain of peroxiredoxin1 (PRDX1), inhibiting its peroxidase activity by driving liquid-liquid phase separation, resulting in the accumulation of lipid peroxidation via the System X_c_^−^-GPX4 axis [16] (Fig. S1).

#### 2.2.2 Non-Canonical Pathways

Both the NAD(P)H-ferroptosis suppressor protein 1 (FSP1)—CoQ_10_ (ubiquinone) axis and the GTP cyclohydrolase 1 (GCH1)–tetrahydrobiopterin (BH4)–dihydrofolate reductase (DHFR) axis are glutathione-independent mechanisms that act in parallel to GPX4 [17]. Myristoylated FSP1 predominantly localizes to the plasma membrane and functions as an NADH/NADPH-dependent oxidoreductase, converting CoQ_10_ (ubiquinone) to CoQ_10_H_2_ (ubiquinol) [18]. CoQ_10_H_2_, as a lipophilic antioxidant, reduces lipid peroxyl radicals to break the chain reaction of lipid peroxidation. In addition, CoQ_10_H_2_ can also indirectly regenerate α-tocopherol (vitamin E) through the CoQ_10_H_2_-mediated electron transport chain (ETC), and dihydroorotate dehydrogenase (DHODH) can also reduce CoQ_10_ to CoQ_10_H_2_ [19], thus inhibiting ferroptosis. Recent studies suggest that FSP1 can function as a vitamin K reductase (VKR), consuming NAD(P)H to reduce vitamin K (VK) to VKH_2_, which acts as a radical-trapping antioxidant [20], but the complete inhibitory mechanism of VK on ferroptosis still requires further elucidation. GCH1 is responsible for catalyzing the initial and rate-limiting reaction in the biosynthesis of BH4, transforming GTP into 7,8-dihydroneopterin triphosphate (NH_2_TP), a key pterin phosphate intermediate. This product is then further metabolized by two additional enzymes, 6-pyruvoyltetrahydropterin synthase (PTS) and sepiapterin reductase (SPR), to ultimately yield BH4 [21]. Seminal work by Mariluz Soula et al. demonstrated that *de novo* BH4 biosynthesis constitutes an enzymatic antioxidant defense system through scavenging lipid peroxides. Mammalian cells employ two complementary mechanisms: the *de novo* pathway maintains basal BH4 levels, whereas the DHFR-mediated salvage pathway becomes essential during cellular BH4 deficiency [22]. Besides, in 2023, the SMURF2 (SMAD-specific E3 ubiquitin protein ligase 2)–GSTP1 (glutathione S-transferase pi 1) axis was found as a new ferroptosis defense way [23]. In summary, additional endogenous antioxidant molecules also exhibit anti-ferroptotic potential. Future research could characterize these molecules to complement the established ferroptosis defense systems.

### 2.3 Metabolic Dependencies

#### 2.3.1 Iron Homeostasis

The maintenance of iron homeostasis depends on precisely regulated iron uptake, storage, utilization, and export mechanisms [24]. Dietary iron is primarily present in the ferric (Fe^3+^) form, which undergoes reduction to the ferrous (Fe^2+^) state for cellular uptake. A coordinated action of intestinal reductases facilitates this critical conversion, most notably duodenal cytochrome B (DCYTB), coupled with subsequent transport via the divalent metal transporter 1 (DMT1) expressed on the apical membrane of duodenal enterocytes. Absorbed Fe^2+^ is exported across the basolateral membrane of enterocytes via ferroportin (FPN), where it undergoes oxidation to Fe^3+^, catalyzed by the multicopper oxidase hephaestin. The resulting Fe^3+^ is subsequently loaded onto transferrin (TF) for systemic delivery to peripheral tissues [25]. This circulating TF-Fe^3+^ is internalized via transferrin receptor 1 (TFR1) and reduced to Fe^2+^ by six-transmembrane epithelial antigen of prostate 3 (STEAP3) in endosomes [26]. Divalent metal transporter 1 (DMT1) transports Fe^2+^ into the cytosol, where it enters the labile iron pool (LIP) (Fig. S2). This pool of redox-active iron plays critical roles in diverse biological processes [25]. Ferritin is a highly conserved iron-binding protein expressed in nearly all nucleated cells, where it functions as the primary intracellular iron storage complex to buffer iron fluctuations [25]. Ferritin can sequester excess iron. However, when its iron-binding capacity is saturated or elevated intracellular iron levels hinder the binding of ferritin heavy chain 1 (FTH1) to nuclear receptor coactivator 4 (NCOA4), which results in ferritinophagy [27,28], the labile iron pool (LIP) exceeds the physiological threshold. Under the condition that iron metabolism-related genes and proteins are dysregulated, iron overload occurs, triggering the fenton reaction.

#### 2.3.2 Lipid Metabolism

The biosynthesis of PUFAs initiates with the conversion of acetyl-CoA to malonyl-CoA catalyzed by acetyl-CoA carboxylase (ACC). Malonyl-CoA serves as the essential two-carbon donor for the fatty acid synthase (FAS)-mediated elongation cycle, followed by desaturation via enzymes (e.g., FADS2 (fatty acid desaturase 2)) to generate PUFAs [6]. The quantity of PUFA-PLs determines the extent of lipid peroxidation and consequently modulates the sensitivity to ferroptosis. The expression levels of ACC, ACSL4, and LPCAT3 directly regulate the abundance of PUFA-PLs. By the way, in 2025, Zhang et al. demonstrated that in lenvatinib-induced ferroptosis of hepatocellular carcinoma (HCC) cells, deltex E3 ubiquitin ligase 2 (DTX2)-mediated degradation of hydroxysteroid (17β) dehydrogenase 4 (HSD17B4) reprograms lipid metabolism by suppressing docosahexaenoic acid (DHA) biosynthesis, thereby attenuating ferroptosis [29]. These findings suggest that HSD17B4 may serve as a novel regulator of lipid metabolism.

In conclusion, advances in ferroptosis mechanisms not only improve the safety evaluation of ferroptosis-targeted clinical translation but also expand the scope of drug discovery. What’s more, ferroptosis-iron overload-WNT/MYC-ornithine decarboxylase 1(ODC1)-polyamine-H_2_O_2_ axis is a positive feedback loop that amplifies ferroptosis and polyamine sensitizes cancer cells to ferroptosis in the way of extracellular vesicles and polyamine supplementation [30]. This discovery suggests a novel strategy to overcome tumor resistance to ferroptosis: using drug combinations to sensitize tumor cells to ferroptosis followed by ferroptosis-inducing therapy.

## 3 Ferroptosis vs. Cuproptosis

Copper is a crucial cofactor in biological systems, but becomes markedly cytotoxic when its concentration exceeds physiological thresholds. Cuproptosis is triggered by excessive copper ions (Cu^2+^) directly binding to lipoylated proteins in the tricarboxylic acid (TCA) cycle, leading to aberrant oligomerization and subsequent formation of cytotoxic insoluble aggregates under prolonged copper stress, which gives rise to the loss of iron-sulfur cluster (ISC) protein, ultimately disrupting mitochondrial respiration chain and causing proteotoxic stress [31]. This does not involve lipid peroxidation and differs from the mechanism triggering ferroptosis.

Dietary copper initially exists in the digestive system as Cu^2+^. Cu^2+^ can be directly absorbed by DMT1 or is first reduced to Cu^+^ by the STEAP family and DCYTB, and then is uptaken by copper transporter 1/2 (CTR1/2) [32]. Due to the non-specific nature of DMT1, it is not suitable as a target for drug development. The absorption of dietary copper mainly relies on CTR1, while dietary iron is primarily absorbed through DMT1. In the intestinal epithelial cells, the copper chaperone antioxidant protein 1 (ATOX1) binds to Cu^+^ and transports Cu^+^ to the opposite side of the epithelial cell [33]. After that, copper-transporting P-type ATPases α (ATP7A) export Cu^+^ into blood circulation [34]. Iron is stored throughout the body cells, while copper is principally concentrated in the liver and eliminated through bile, so the metabolic imbalance of copper ions is closely related to liver and gallbladder diseases [35,36]. With the assistance of circulating chaperone proteins, copper is distributed to other tissues or organs, and then chiefly taken up by CTR1 [37].

In cells, copper is stored by metallothionein (MT) and other ligands [38]. GSH does act as one of the ligands, binding copper to control and regulate copper homeostasis. This mechanism establishes a shared molecular barrier and represents a critical molecular intersection between ferroptosis and cuproptosis. Buthionine sulfoximine (BSO), a specific inhibitor of GSH biosynthesis, has been proven to induce both ferroptosis and cuproptosis [39,40]. Copper chaperone for superoxide dismutase (CCS) can transport free copper to bind with superoxide dismutase 1 (SOD1), which is located between the cytosol and the mitochondrial intermembrane space (IMS) [41]. Cytochrome oxidase 17 (COX17) and copper ligands (CuL) can help copper enter the mitochondria [42,43].

Copper ionophores that can help increase intracellular copper levels are useful tools to study copper toxicity or induce cuproptosis [5]. Mitochondrial ferredoxin 1 (FDX1) and protein lipoylation are two important regulators of copper ionophore-induced cell death. FDX1 functions as a reductase that converts Cu^2+^ to its more toxic Cu^+^ form and causes the destabilization of Fe-S cluster proteins [44]. It can serve as a direct molecular target of elesclomol, which is also an upstream regulator of protein lipoylation [45]. Key genes regulating protein lipoylation comprise: (1) Lipoic acid biosynthesis components (*LIAS* (lipoyl synthase), *LIPT1* (lipoyl amidotransferase), *DLD* (dihydrolipoyl dehydrogenase)); (2) Lipoylated subunits of pyruvate dehydrogenase (PDH) complex (*DLAT* (dihydrolipoyl transacetylase), *PDHA1* (PDHα), *PDHB* (PDHβ)), as protein targets of lipoylation [46]. Similar to ferroptosis, dysregulation of copper metabolism also plays a dual role in cancer progression [32]. On the one hand, superfluous copper, as a cofactor for various enzymes, satisfies the conditions for the rapid proliferation of tumors [47,48]. On the other hand, copper suppresses cancer development by binding inappropriately with functional proteins [49,50] (Table 1).

**Table 1:** Differences between ferroptosis and cuproptosis

Category	Ferroptosis	Cuproptosis
**Key ion**	Fe^2+^ [3]	Cu^2+^
**Metal carriers**	TF, Ferritin	Circulating chaperone proteins, MT, GSH
**Morphological features**	Mitochondrial size reduction Decreased number of mitochondrial cristae Increased mitochondrial membrane density	Mitochondrial atrophy Cell membrane rupture Endoplasmic reticulum damage Chromatin damage
**Core mechanism**	Lipid peroxidation [38]	Lipoylated protein aggregation
**Key regulators**	ACSL4, LPCAT3, SystemX_c_, GPX4	FDX1, LIPT1, LIAS, DLD, DLAT, PDHA1, PDHB
**Inhibitors**	Ferrostatin-1, ML162	Copper chelator (e.g., GSH)
**Inducer**	Erastin, RSL3	Copper ionophores (e.g., elesclomol) [39]
**Cell death markers**	Fe^2+^, ROS, GSH, PL-OOHs, Hyperoxidized PRDX3 (chronic liver diseases) [51], GPX4, ACSL4, MDA, 4-HNE	Cu^+^, SLC31A1, ATP7A/B, FDX1, LIAS, GSH, DLAT, DLST, Fe–S cluster proteins [5,52]
**Related disease models**	Parkinson’s disease [53], kidney disease [54], Alzheimer’s disease [55], Acute lung injury/acute respiratory distress syndrome [56]	Chronic obstructive pulmonary disease [57], non-alcoholic fatty liver disease [58], Alzheimer’s disease [59], rheumatoid arthritis [60]

Note: LIPT1, lipoyltransferase 1; LIAS, lipoic acid synthase; DLD, dihydrolipoamide dehydrogenase; DLAT, dihydrolipoamide S-Acetyltransferase; PDHA1, pyruvate dehydrogenase E1 subunit alpha 1; PDHB, pyruvate dehydrogenase E1 subunit beta; PRDX3, peroxiredoxin 3; DLST, dihydrolipoyllysine-residue succinyltransferase; MDA, malondialdehyde; 4-HNE, 4-hydroxynonenal.

**Figure 2:**
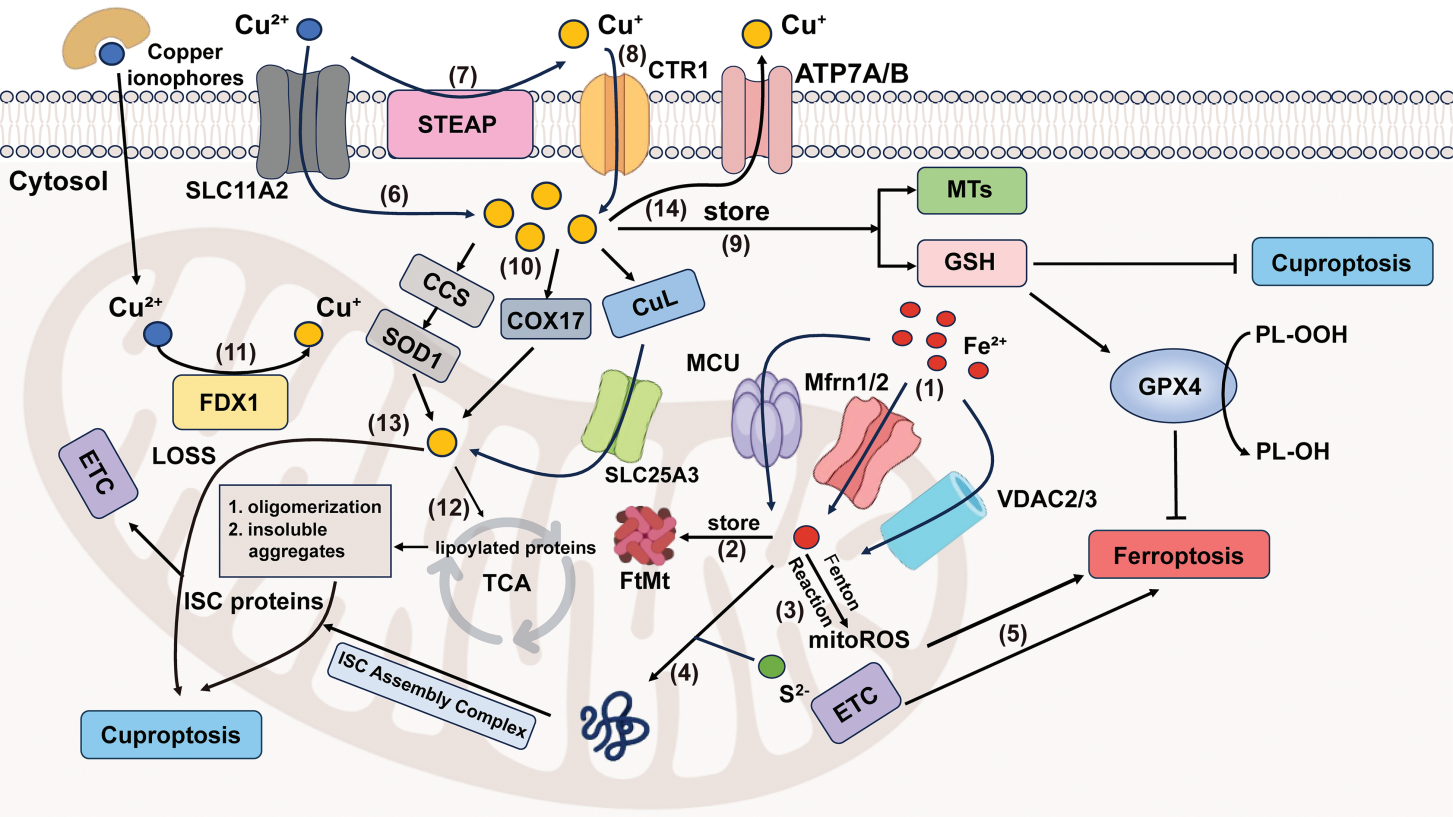
Crosstalk between ferroptosis and cuproptosis. (1) MCU, Mfrn1/2, VDAC2/3 transport Fe^2+^ into mitochondrial matrix. (2) FtMt stores Fe^2+^ in mitochondria. (3) Fe^2+^ in mitochondria can generate mitoROS through the Fenton reaction. (4) Mitochondrial Fe^2+^ and S^2−^ enter the ISC assembly complex, responsible for the maturation of all ISC proteins, which is an important component of ETC. (5) ETC electron leakage and mitoROS participate in ferroptosis progression. (6) Cu^2+^ can enter cells either by forming lipid-soluble complexes with copper ionophores or through SLC11A2-mediated transport. (7) The STEAP family can reduce Cu^2+^ to Cu^+^. (8) Cu^+^ is specifically transported into cells by CTR1, a high-affinity copper importer. (9) In cells, Cu^+^ can be stored by MTs and GSH. (10) Mitochondria take up Cu^+^ through CCS-SOD1, COX17 (shuttling between the cytoplasm and the mitochondria) and CuL-SLC25A3 ways. (11) Cu^2+^ in mitochondria is reduced to the more toxic form (Cu^+^) by FDX1. (12) Excessive Cu^+^ induces lipoylated proteins’ oligomerization, and consequently, forms insoluble aggregates, ultimately causing cuproptosis. (13) Cu^+^ is also involved in cuproptosis by affecting the stability of ISC proteins. (14) Excess copper is exported extracellularly by ATP7A/B transporters. Abbreviation: SLC11A2, Solute Carrier Family 11 Member 2; ATP7A/B, ATPase copper transporter 7A and 7B. Some of the materials in the picture are taken from the drawing software “Biorender”

Mitochondria play an important role in various physiological processes, like energy production [61]. Ferroptosis can occur either with or without the involvement of mitochondria, but cuproptosis must be mitochondria-dependent. When either of them occurs, the morphology of the mitochondria will change [5,62]. However, there are still differences between them. Mitochondria are the main source of ROS and major sites of iron utilization (containing up to 20%–50% within the cell) [63,64]. The entry of Fe^2+^ into mitochondria mainly depends on mitochondrial calcium uniporter (MCU), mitoferrin 1/2 (Mfrn1/2), and voltage-dependent anion-selective channel 2/3 (VDAC2/3). The way of generating mitochondrial reactive oxygen species(mitoROS) is similar to that of the cytoplasm. Namely, it also has ferritin (FtMt, an H-type ferritin) used in storage, and a free iron pool, which undergoes the Fenton reaction under abnormal conditions [65–67]. And another exclusive way is electron leakage of the ETC [64]. Fe-S cluster proteins are a significant component of ETC, and precisely need to undergo a mature process through the mitochondrial iron entering ISC assembly machinery [68]. Disruption of Fe-S clusters may cause electron leakage in the mitochondrial ETC, promoting ROS generation. In cuproptosis, it is excessive copper that leads to protein changes that damage the integrity of mitochondria, different from lipid peroxidation. Cu^2+^ enters mitochondria through copper ionophores, while Cu^+^ is transported via multiple specialized pathways, including the CCS-SOD1 chaperone system, COX17-mediated shuttling between the cytoplasm and mitochondria, and the CuL-Solute Carrier Family 25 Member 3 (SLC25A3) carrier complex in the mitochondrial inner membrane (Fig. 2). As previously mentioned, copper ions (Cu^+^/Cu^2+^) accumulation leads to the degradation of Fe-S cluster proteins. From the above, we can observe that Fe-S cluster proteins also seem to be the intersection point of ferroptosis and cuproptosis. So here comes the question: Can ferroptosis induced by iron overload occur simultaneously with cuproptosis? The answer is yes. Based on the above discussion, we can conclude that excessive copper that induces cuproptosis can first destroy Fe-S cluster proteins, thereby affecting the ETC, generating ROS, and promoting ferroptosis. Ferroptosis can help consume GSH and promote cuproptosis. Recent research also supplements and proves this point, which indicates that lactate metabolism plays a critical role in regulating both ferroptosis and cuproptosis [69]. Lactate accumulation induces intracellular acidification, creating a dual regulatory effect on metal metabolism. The resulting acidic environment directly promotes FTH1 dissociation to release stored iron, consequently elevating intracellular iron levels. Simultaneously, lactate-mediated suppression of glycolysis reduces cellular ATP availability, which functionally impairs the copper export activity of ATP7B (copper transporting ATPase 7β) and leads to progressive copper retention. This coordinated disruption of iron and copper homeostasis may synergistically potentiate metal-dependent cell death pathways. A mitochondria-targeted nano-heterojunction platform (MIL-Cu_1_._8_S-TPP/FA) delivers copper and iron ions simultaneously, enhances ROS catalytic activity, disrupts mitochondrial iron-sulfur proteins, and synergistically induces ferroptosis and cuproptosis, achieving potent anti-tumor therapy combined with photothermal effects [70].

At present, there are not enough articles published regarding the specific concentration thresholds for iron and copper, nor has there been a comparison of the temporal kinetics of cuproptosis and ferroptosis. However, based on the mechanism and the available data, it can be inferred that the amount of iron required to cause ferroptosis is greater than that for cuproptosis and the time required to induce ferroptosis in the same type of cells may be longer than that for cuproptosis. Initially, ferroptosis is a process that requires gradual accumulation and collapse. But when copper levels are excessive, it causes direct damage to the mitochondria, bringing about cell death. Secondly, under most normal physiological conditions, the iron content in mammalian cells is significantly higher than the copper content [71,72]. Eventually, according to the data, for example, ferric ammonium citrate (FAC) exhibits cytotoxic effects on HT-1080 fibrosarcoma cells only at concentrations ≥5 mM *in vitro* [73], but in HEK293T cells overexpressing solute carrier family 31 member 1 (SLC31A1), a concentration of 10–20 μM was sufficient to induce cuproptosis in the majority of cells, with a significant decrease in viability approaching 0 [5]. But due to limited current clinical data and model complexity, the iron-copper concentration threshold awaits further verification in subsequent research.

In the first half of 2025, research and development of antitumor drugs based on cuproptosis primarily focused on nanotechnology. The developed tussah silk fibroin nanoparticles (TSF@ES-Cu NPs) effectively protect elesclomol (ES) and Cu^2+^, successfully addressing the short blood half-life issue of ES. The system innovatively utilizes the targeting function of natural RGD (Arginine-Glycine-Aspartic acid) peptides and the unique secondary structure characteristics of TSF, significantly enhancing drug-specific enrichment and controlled release in pancreatic tumor tissues, ultimately achieving remarkable improvement in therapeutic efficacy [74]. The constructed copper-based Prussian blue nanostructure (NCT-503@Cu-HMPB) selectively induces tumor cell cuproptosis by simultaneously inhibiting serine metabolism and enhancing copper ion toxicity, establishing a novel paradigm for cancer therapy based on cuproptosis and metabolic reprogramming [75]. Triple-negative breast cancer (TNBC) develops drug resistance by resisting cuproptosis through AKT1-mediated FDX1 phosphorylation [76]. The combination of AKT1 inhibitors with copper ionophores synergistically suppresses TNBC tumorigenesis, suggesting a potential novel therapeutic strategy for TNBC. A new ROS-responsive targeting cuproptosis nanomedicine (CuET@PHF) overcomes hypoxia via photothermal effects, upregulates FDX1, and triggers cuproptosis in TNBC stem cells (CSCs) for the first time, synergizing with immunogenic cell death to suppress tumor growth, recurrence, and metastasis [77]. In addition, the natural fungal-derived pochonin D (PoD) inhibits peroxiredoxin-1 (PRDX1) enzymatic activity by targeting Cys173, modulates cuproptosis, and serves as a novel copper ionophore and therapeutic strategy for TNBC [78].

## 4 Ferroptosis in Cancers

### 4.1 The Roles of Ferroptosis in Cancer

Ferroptosis exerts bidirectional regulatory effects in oncogenesis, functioning as a tumor-suppressive mechanism in certain contexts while unexpectedly facilitating tumorigenesis in others. For instance, the combinatorial treatment of temsirolimus (mTOR inhibitor) and RSL3 (GPX4 inhibitor) synergistically induces ferroptosis, resulting in significant restraint of liver cancer progression [79]. Gambogenic acid inhibits the malignant progression of non-small cell lung cancer (NSCLC) by inducing GCH1-regulated ferroptosis [80]. Metformin suppresses tumor growth by enhancing ferroptosis in ovarian cancer cells under energy stress conditions [81]. However, ferroptosis can also promote tumor progression under certain circumstances, which is related to the tumor microenvironment (TME) (triggering inflammation-related immunosuppression) and changes in key genes (e.g., p53). The inflammatory response associated with ferroptosis contributes to the formation of a tumor-promoting microenvironment through multiple mechanisms: stimulating cancer cell proliferation and migration, promoting angiogenesis, and suppressing adaptive immune function. Specifically, damage-associated molecular patterns (DAMPs) released during ferroptosis induce the secretion of inflammatory cytokines such as interleukin-8 (IL-8) as well as recruit and activate various immune cells (e,g., tumor-associated macrophages, myeloid-derived suppressor cells, regulatory T cells), directly promoting cancer cell proliferation, while upregulating pro-angiogenic factors like vascular endothelial growth factor (VEGF) to accelerate tumor vascularization [82]. More importantly, the inflammatory microenvironment triggered by ferroptosis significantly inhibits T cell activation and proliferation, thereby compromising the clinical efficacy of immunotherapy [83]. When ROS levels are elevated, inflammatory cytokines and ROS upregulate the expression of ferroptosis-inducing enzymes (e.g., ACSL4), induce iron overload and membrane lipid peroxidation, thereby rendering cancer cells more susceptible to ferroptosis [84,85]. p53 is closely associated with the ferroptosis metabolic pathway and serves as its key regulatory factor [86]. When lipid peroxidation damage is mild and repairable, p53 helps cells resist ferroptosis by inhibiting dipeptidyl peptidase-4 (DPP4) activity or upregulating CDKN1A/p21 expression [87]. If the damage persists or becomes too severe, p53 induces ferroptosis by suppressing SLC7A11 expression or promoting spermidine/spermine N1-acetyltransferase 1 (SAT1) and glutaminase 2 (GLS2) expression, thereby eliminating damaged cells [88]. For example, ferroptotic cancer cell-derived 8-hydroxyguanosine (8-OHG) activates the stimulator of interferon genes (STING)-dependent DNA sensing pathway in tumor-associated macrophages, thereby fostering a pro-inflammatory TME that promotes pancreatic adenocarcinoma development [89,90]. Acidic nuclear phosphoprotein 32Et (ANP32Et) promotes esophageal cancer progression via p53/SLC7A11 axis-regulated ferroptosis [91]. This phenomenon may also be closely associated with intracellular ROS levels. Studies have shown that low ROS concentrations exert protective effects on normal cells, whereas moderate ROS levels, while detrimental to normal cells, may promote tumor cell proliferation. Nevertheless, excessively high ROS accumulation can ultimately induce tumor cell death [92,93]. For these cancer types where ferroptosis plays a promoting role, the use of ferroptosis inhibitors may provide certain alleviating effects. The combination of ferroptosis inhibitors with other antitumor drugs might achieve better therapeutic outcomes. However, specific treatment regimens should be tailored based on TME characteristics and tumor genetic mutation profiles. Regarding the carcinogenic effects and mechanisms of iron, the overall picture is inadequate, and more research is still needed.

Meanwhile, different types of cancer have varying sensitivities to ferroptosis, primarily determined by specific metabolic signatures and the integrity of antioxidant defense systems. It is reported that the glutamine-dependent breast cancer (BC) subgroup is sensitive to ferroptosis, and this sensitivity is associated with ACSL4 expression [94]. TNBC with high LncFASA expression exhibits enhanced susceptibility to ferroptosis [16]. While certain cancer types exhibit marked susceptibility to ferroptosis, others demonstrate intrinsic or acquired resistance mechanisms. In the lymphatic microenvironment, oleic acid protects melanoma cells from ferroptosis in an ACSL3-dependent manner, substantially enhancing their metastatic tumor-forming capacity. Compared to subcutaneously implanted melanoma, these lymph-colonizing cells exhibit superior ferroptosis resistance [95]. In lung cancer cells, GSH represents the most abundant antioxidant. The production of GSH is regulated not only by System X_c_^−^ but also involves the participation of glutamate transporters, particularly the excitatory amino acid transporter 3 (EAAT3). In 2025, Wen et al. reported that NF-κB drives EAAT3 expression, and knockdown of EAAT3 enhances the susceptibility of lung cancer cells to ferroptosis. Furthermore, upregulated EAAT3 in NSCLC tissues correlates with p65 protein levels, while smoking-induced inflammation promotes EAAT3 expression in lung cancer models [96]. Subsequent research could focus on targeting the NF-κB/EAAT3 axis in lung cancer to explore combined ferroptosis therapies. In 3D-cultured colorectal cancer models, the hybrid epithelial–mesenchymal transition (EMT) phenotype confers robust ferroptosis resistance through integrin mechanotransduction and mitochondrial metabolic remodeling. Notably, this protective phenotype emerges as a delayed consequence of E-cadherin loss, rather than the anticipated ferroptosis sensitization [97]. Cancer-associated fibroblasts (CAFs) confer doxorubicin resistance to TNBC by augmenting zinc finger protein 64 (ZFP64)-mediated histone lactylation, thereby modulating ferroptosis sensitivity [98].

Emerging evidences reveal that ferroptosis induction may not only prove therapeutically ineffective in certain malignancies but could paradoxically promote tumor progression. Therefore, clinical application of ferroptosis inducers necessitates rigorous molecular classification and susceptibility assessment of the target cancer type.

### 4.2 Targeting Ferroptosis in Cancer: Drug/Compounds and Therapy

Current research has found that certain active components in traditional Chinese herbal medicine can influence the initiation and progression of cancer by regulating the ferroptosis pathways. Tanshinone IIA (Tan IIA) induces ferroptosis in BC cells by modulating the lysine-specific demethylase 1 (KDM1A)-protein inhibitor of activated STAT 4 (PIAS4) axis to regulate SUMOylation-dependent destabilization of SLC7A11 [99]. In addition, Tanshinone IIA has been found to promote ferroptosis in cutaneous melanoma via STAT1-mediated upregulation of prostaglandin-endoperoxide synthase 2 (PTGS2) expression [100] and in colorectal cancer (CRC) through the suppression of SLC7A11 expression via the PI3K/AKT/mTOR pathway [101]. However, Li et al. reported that it inhibits hydrogen peroxide-induced ferroptosis in melanocytes by activating the nuclear erythroid 2-related factor 2 (Nrf2) signaling pathway [102]. Nanoparticles related to Tanshinone IIA for anticancer applications will be discussed in the nanotechnology section. Although Tanshinone IIA has entered clinical trials for diseases such as left ventricular remodeling secondary to acute myocardial infarction [103], lipopolysaccharides (LPS)-induced mastitis [104], and cardiac function recovery, no cancer-related clinical trials have been identified to date [105]. Erianin inhibits CRC progression by inducing ferroptosis through promoting GPX4 ubiquitination and degradation [106]. Matrine induces ferroptosis in cervical cancer (CC) by activating the Piezo1 channel, which subsequently downregulates GPX4 expression [107] (Table 2). Similar to the clinical trial progress of Tanshinone IIA, matrine has been investigated in clinical trials for conditions such as perianal infection after chemotherapy for acute leukemia [108], chronic hepatitis B [109], and silicosis [110], but no cancer-related clinical trials have been identified to date. Future research may employ extraction, identification and chemical modification of bioactive compounds from Chinese herbs and other medicinal plants to screen for novel anticancer agents, whose mechanisms of action may involve but are not limited to the ferroptosis pathway. At present, there are very few traditional Chinese medicine components that have entered clinical trials. It is hoped that relevant clinical tests can be conducted in the future.

**Table 2:** Ferroptosis-related drugs/compounds classified by targets

Target	Drugs/Compounds	Indications (Examples)	Development Stage	Refs.
**GCH1 inhibition**	Gambogenic Acid	NSCLC	Preclinical	[80]
**ACSL4 modulation**	Tubuloside A	CRC	Preclinical	[111]
**System Xc**^**−**^ i**nhibition**	Erastin	HCC, Melanoma, NSCLC, LUAD, Ovarian cancer, Anaplastic thyroid cancer	Preclinical	[3]
	Tanshinone IIA	BC, cutaneous melanoma, colorectal cancer	Preclinical	[99–101]
	CSIR	OC	Preclinical	[112]
	Sulforaphane	OC	Preclinical	[113]
	FTD/TPI	CC	Approved	[114]
**GPX4 inhibition**	Erianin	CRC	Preclinical	[106]
	Matrine	CC	Preclinical	[107]
	RSL3	Neuroblastoma, Osteosarcoma, Lung cancer, Rhabdomyosarcoma, BC	Preclinical	[115]
	Fatostatin	GBM	Preclinical	[116]
	α-Hederin	TNBC	Preclinical	[117]
	HMPB@ERY@PT	OC	Preclinical	[118]
**FSP1 inhibition**	Temsirolimus	Liver cancer	Preclinical	[79]
	Sorafenib	Liver cancer, Kidney cancer, Thyroid cancer	Approved	[119]
**PUFA metabolism**	Imetelstat	AML	Approved	[120]
	Alkannin	GC	Preclinical	[121]
**Iron overload**	CNSI-Fe (II)	Advanced Solid Tumor	Phase I	[122]
**Ferritinophagy induction**	Baicalin	bladder cancer	Preclinical	[123]
	HP and TMZ	GBM	Phase II	[124]

Note: CSIR, SRF@CuSO_4_·5H_2_O@IR780; FTD/TPI, trifluridine/tipiracil; HP, haloperidol; TMZ, temozolomide; NSCLC, non-small cell lung cancer; CRC, colorectal cancer; HCC, hepatocellular carcinoma; LUAD, lung adenocarcinoma; BC, breast cancer; OC, osteosarcoma; CC, cervical cancer; GBM, glioblastoma; AML, acute myeloid leukemia; GC, gastric cancer.

Imetelstat promotes the formation of PUFA-PLs, effectively ameliorating acute myeloid leukemia (AML) [120]. Researchers identified alkannin as a clinically promising natural product against gastric cancer (GC), and elucidated a previously unreported ferroptosis mechanism on lipid homeostasis regulation via the c-Fos/sterol regulatory element binding transcription factor 1 (SREBF1) axis [121]. Mechanistically, priming with oxidative stress-inducing chemotherapy potentiates the susceptibility of tumors to ferroptosis inducers, proposing a novel combinatorial therapeutic paradigm.

Exploring ferroptosis inducers can help establish an upstream and downstream relationship network. Nuclear accumulation of p53 and enhanced p62/SLC7A11 protein-protein interaction can downregulate SLC7A11, found through ferroptosis inducers (sulforaphane and Trifluridine/Tipiracil (FTD/TPI)) correlational studies [113,114]. In the experiment where Sorafenib induces ferroptosis, it is found that Sorafenib achieves this effect by promoting the ubiquitination and degradation of FSP1 mediated by tripartite motif containing 54 (TRIM54) [119]. Meanwhile, the finding of ferroptosis is beneficial in revealing the unknown mechanism of anti-cancer drugs. Originally, FTD/TPI just had promising anticancer activity, but its anticancer targets remained incompletely understood. Experimental data have manifested that it is associated with ferroptosis [114]. Mori Folium has anticancer activity, but its underlying mechanisms were unknown at first; it has now been revealed that it induces ferroptosis in GC cells by modulating the PI3K/AKT/GSK3β/NrF2 signaling pathway [125]. Besides, Alkannin [121], Tubuloside A (a proline 4-hydroxylase, alpha polypeptide III (P4HA3)) degrader, providing a potential strategy to overcome CRC resistant varieties) [111] are the same, elucidating the undefined mechanisms of action of anticancer agents.

Combination therapy has shown remarkable advantages in the treatment of cancer. Many studies indicate that when ferroptosis inducers are combined with conventional anticancer drugs, they not only achieve multi-target synergistic effects, but existing experimental data also confirm that this combination strategy can significantly enhance antitumor efficacy. When Baicalin was combined with 5-Fu to combat GC, the expression levels of TFR1, nicotinamide adenine dinucleotide phosphate oxidase 1 (NOX1), and COX2 were upregulated, whereas those of FTH1, ferritin light chain (FTL), and GPX4 were downregulated, leading to superior therapeutic efficacy [92]. Haloperidol (HP), as an effective adjunct therapy, reverses Temozolomide **(**TMZ) resistance and improves chemoradiotherapy efficacy in glioblastoma (GBM) [124]. Propranolol and capecitabine synergistically induce ferroptosis in CRC, although the original study demonstrated efficacy only in *BRAF* (V600E)-mutant HT-29 cells [126] (Table 3). Simultaneous induction of multiple RCD pathways, such as apoptosis and ferroptosis, represents a promising approach to augment anticancer efficacy and circumvent resistance mechanisms. With the discovery of ferroptosis regulatory factors in cancer and chemotherapy drug resistance targets, such as transmembrane protein 160 (TMEM160) [127], syntaxin 1A (STX1A) [128], vimentin-Interacting protein AS39 (VIPAS39) [129], and serine/threonine-protein kinase (SIK1) [130], the expansion of the target range has also broadened the application of combined treatment. Inhibit the known targets that confer resistance to ferroptosis for specific cancer types, and then induce ferroptosis, and so on. The mechanisms of action of drugs related to ferroptosis mainly focus on their core mechanisms, while the research on the development of drugs targeting these drug-resistant-related targets is relatively scarce.

**Table 3:** Ferroptosis-related drugs/compounds acting on multiple targets

Drugs	Mechanisms	Indications (Examples)	Development Stage	Refs.
Mori Folium ethanol extracts	ROS and MDA production, promoting iron accumulation, downregulating GPX4 and xCT, GSH depletion	GC	Preclinical	[125]
4-MD	inhibit DNMT1, SLC7A11, GPX4 expression	lung cancer	Preclinical	[131]
MnO2R@FePDAc	GPX4/FSP1 pathways inhibition, ROS production, GSH depletion	OSCC (*in vitro*)	Preclinical	[132]
USINAs(131I-aPD-L1)	SLC7A11 downregulation, GPX4 inhibition, ROS production, GSH depletion	murine breast cancer 4T1 cells (*in vitro*)	Preclinical	[133]
Aur/Plu@HM	antioxidant molecules depletion, deprive the metal cofactor of SCD1	ovarian cancer	Preclinical	[134]
PCO	catalyze the Fenton-like reaction, displays dual peroxidase- and glutathione oxidase-mimic enzymatic activity, GSH depletion, GPX4 downregulation	CRC	Preclinical	[135]
DP-HBN/RA	ROS production, inactivation of GPX4, exacerbate irreparable DNA damage and release DNA fragments that activate the cGAS-STING signal pathway	TNBC	Preclinical	[136]
Auriculasin	mitochondrial oxidative stress, inhibiting PI3K/Akt pathway	NSCLC	Preclinical	[137]
Fucoxanthin	triggering PANoptosis (apoptosis, necroptosis, pyroptosis) and ferroptosis (AMPK/Nrf2/HMOX1 pathway)	Ovarian Cancer	Preclinical	[138]
Metformin	upregulating ferroptosis-related genes, downregulating GPX4 and SLC7A11, upregulating RBMS3, increased MDA and Fe^2+^ levels, reduced GSH	Ovarian Cancer	Preclinical	[139]
Icariin	binding with HMGA2, STAT3, and HIF-1α proteins, ROS and MDA production, promoting iron accumulation, GSH depletion, downregulating GPX4 and SLC7A11	CRC	Preclinical	[140]
PD2	mitochondrial damage, mtROS production	BC	Preclinical	[141]
DP	HMOX1 activation, downregulation of the SLC7A11/GSH/GPX4 axis	CRC	Preclinical	[142]
Sorafenib, SBRT	SBRT: inhibite SLC7A11 expression, impeding the phosphorylation of JNK and c-Jun and the transcription of NRF2 Sorafenib: targeting SLC7A11	CRC, liver metastasis	Phase II	[143]
MLN4924, belinostat	quantitative increases in the oxidative stress protein NQO1, ferroptosis protein SLC7A11, and GSR	relapsed/refractory AML, myelodysplastic syndrome	Phase I	[144]

Note: 4-MD, 4-methoxydalbergione; DNMT1, DNA methyltransferase 1; SLC7A11, solute carrier family 7 member 11; OSCC, oral squamous carcinoma; SCD1, stearoyl-CoA desaturase 1, its upregulation leads to resistance to ferroptotic therapy in ovarian cancer; MDA, malondialdehyde; xCT, system X_c_^−^; PD2, platycodin D2; DP, dracorhodin perochlorate; SBRT, stereotactic body radiotherapy; JNK, c-jun N-terminal kinase; NrF2, nuclear factor erythroid 2-related factor 2; NQO1, quinone oxidoreductase 1; GSR, glutathione reductase.

In the development of ferroptosis-based cancer therapeutics, achieving selective cytotoxicity against cancer cells while sparing normal cells remains a central challenge. In recent years, nanomaterials with superior targeted delivery properties have been extensively investigated and applied due to their ability to enhance therapeutic specificity and safety significantly. Carbon nanoparticles-Fe (II) Complex (CNSI-Fe (II)) effectively delivers iron into tumor cells while exhibiting minimal systemic toxicity in tumor-bearing mice [122], having now advanced to Phase I clinical trials. Carbon nanosphere suspension injection (CNSI) is a commercially available and clinically applied carbon nanomaterial [145]. Animal studies and clinical observations indicate that CNSI exhibits minimal systemic toxicity [146] and contains abundant oxygen-containing functional groups capable of interacting with Fe^2+^. As a biocompatible carbon material, CNSI does not induce oxidative stress [147]. When administered via intratumoral injection, the limited quantity of TRF and other transport proteins restricts Fe^2+^ translocation into the cytoplasm. Consequently, neither CNSI nor iron alone demonstrates significant efficacy in tumor growth suppression. The potent antitumor effect of CNSI-Fe is attributed to the synergistic interaction between CNSI and Fe^2+^. Cellular uptake of CNSI particles via endocytosis facilitates Fe^2+^ delivery into the cytoplasm. Fe^2+^ exerts cytotoxicity by efficiently catalyzing H_2_O_2_ decomposition, while the elemental carbon further enhances the fenton reaction. Due to the blockage of the FPN pathway in tumor cells, Fe^2+^ cannot be effluxed, leading to its prolonged intracellular accumulation. Experimental studies confirm that the majority of CNSI and Fe remains localized within the tumor tissue post-injection. Since the tumor is surgically resected, long-term toxicity is unlikely to occur. From the perspective of targeted delivery mechanisms, CNSI-Fe(II) is particularly suitable for solid tumors. Eriodictyol-cisplatin nanomedicine synergistically induces ferroptosis and enhances chemosensitivity in osteosarcoma (OC) cells [118]. A carrier-free nanomedicine SRF@CuSO_4_·5H_2_O@IR780 (CSIR) synergistically enhances OC tumor eradication through GSH depletion, photodynamic therapy (PDT), and photothermal therapy (PTT), providing a multi-modal therapeutic approach [112]. The exploration of nanotechnology and related precision delivery approaches may expand the repertoire of applicable ferroptosis inducers or inhibitors for disease treatment. pH-responsive Tanshinone IIA-loaded calcium alginate nanoparticles overcome the poor aqueous solubility of Tanshinone IIA, thereby addressing its clinical application limitations [148]. Moreover, nanotechnology can also serve as a strategy for combination therapy. Arsenene-Vanadene nanodots (AsV) drug delivery system combines arsenic (As)-induced apoptosis with vanadium (V)-enhanced ferroptosis, opening new avenues for TNBC immunotherapy [149]. Redox-driven hybrid nanozyme (M@GOx/Fe-HMON) dynamically activates either ferroptosis or disulfidptosis in HCC by adapting to glucose heterogeneity [150]. Under high glucose, it triggers Gox glucose oxidase (Gox)-peroxidase (POD) cascade reactions to induce ferroptosis via ROS and GSH depletion, while under low glucose, it promotes disulfidptosis via NADPH exhaustion and cystine metabolism disruption, finally amplifying antitumor efficacy. A novel stimulus-responsive (pH/GSH-responsive) antitumor nanoplatform (ISL@RLP-Fe), based on polysaccharide-flavonoid-iron complexes, integrates the immunostimulatory activity of natural macromolecule *Rosa laevigata* polysaccharide (RLP), the chemotherapeutic potential of isoliquiritigenin (ISL), and the chemodynamic therapy (CDT) functionality of iron ions [151]. This system enables tumor microenvironment-specific disintegration and drug release, significantly reducing tumor mass and inhibiting cancer cell migration in preclinical models. The study demonstrates groundbreaking innovation by integrating traditional Chinese herbal medicine components with advanced nanotechnology, thereby establishing a novel methodology and conceptual framework for applying natural phytochemicals in anticancer therapeutics.

## 5 Conclusions and Future Prospects

Given the growing challenge of drug resistance to conventional therapies, ferroptosis induction has emerged as a promising novel approach and strategic alternative for clinical cancer treatment. Nonetheless, numerous compounds with clinical potential remain in the experimental stage, and their therapeutic applications require further validation. Ferroptosis-targeted anticancer therapies confront the same resistance challenges. The main directions that can be further expanded at present are as follows: (1) The crosstalk between ferroptosis and cuproptosis suggests potential for combinatorial targeting. Future therapeutic strategies could leverage this synergy by designing bifunctional agents that concurrently engage both mechanisms, thereby maximizing tumoricidal efficacy while minimizing off-target effects. (2) In comparative studies of ferroptosis and cuproptosis, the kinetic profiles (including initiation time and progression rate) and the minimum effective concentrations of copper/iron ions required to induce cell death still lack systematic quantitative analysis. (3) Multiple studies in 2025 have reported that traditional Chinese herbal medicines can inhibit cancer by regulating the ferroptosis pathway. These findings not only suggest the potential to extract, identify, and chemically modify their bioactive compounds, but also help elucidate the specific molecular mechanisms underlying their therapeutic effects. (4) Targeted drug delivery to tumor sites is essential to minimize damage to normal cells and expand therapeutic applications. Therefore, nanotechnology can be leveraged to develop more ferroptosis-inducing anticancer agents. (5) Combination therapeutic strategies still hold potential for further development, which may provide innovative solutions to address drug resistance challenges and reduce medication toxicity.

## Supplementary Materials

Figure S1 Concise schematic of ferroptosis defense mechanism. Some of the materials in the picture are taken from the drawing software "Biorender". (1) System Xc− imports cystine by exchanging intracellular glutamate (1:1). (2) Cystine is then reduced to cysteine. (3) GPX4 uses GSH to detoxify lipid hydroperoxides, suppressing lipid peroxidation. (4) USP8 stabilizes GPX4 through deubiquitination. (5) zDHHC8 mediates GPX4 palmitoylation at Cys75 to suppress ferroptosis. (6) PRDX1 suppresses ferroptosis by scavenging peroxides, whereas LncFASA inactivates PRDX1 through phase separation, leading to unchecked lipid peroxidation. (7) FSP1 generates ferroptosis-suppressive CoQ10H2 from CoQ10. (8) FSP1 reduces vitamin K to VKH2 using NAD(P)H, generating a radical-trapping antioxidant. (9) GCH1 converts GTP into NH2TP. (10) PTS and SPR work coordinately to produce BH4, which constitutes an enzymatic antioxidant defense system.

Figure S2 Simplified schematic diagram of the mechanism of ferroptosis. Some of the materials in the picture are taken from the drawing software "Biorender". (1) ACC catalyzes acetyl-CoA conversion to malonyl-CoA. (2) ACSL4 catalyzes the acylation of PUFAs with CoA, generating PUFA-CoAs. (3) LPCAT3 then incorporates PUFA-CoAs into PLs, forming PUFA-PLs. (4) LOXs and POR can initiate lipid peroxidation by catalyzing PUFA dioxygenation. (5) Free iron triggers Fenton reaction, generating excessive ROS. (6) TF-Fe^3+^ is taken up by TFR1, reduced to Fe^2+^ by STEAP3 in endosomes, and transported to the cytosol via DMT1, joining the LIP. (7) Ferritin sequesters iron but undergoes ferritinophagy, releasing stored iron into the LIP. (8) Excess Fe^2+^ in the LIP drives fenton reaction, triggering lipid peroxidation.

## Data Availability

Data sharing not applicable to this article as no datasets were generated or analyzed during the current study.

## Supplementary Materials

The supplementary material is available online at https://www.techscience.com/doi/10.32604/or.2025.069049/s1.
