# Supplementary figures and images for: Ferroptosis: Mechanisms, Comparison with Cuproptosis and Emerging Horizons in Therapeutics
Source: Oncol Res. 2025 Dec 30;34(1):8. doi: 10.32604/or.2025.069049 (PMC12774555; doi:10.32604/or.2025.069049)

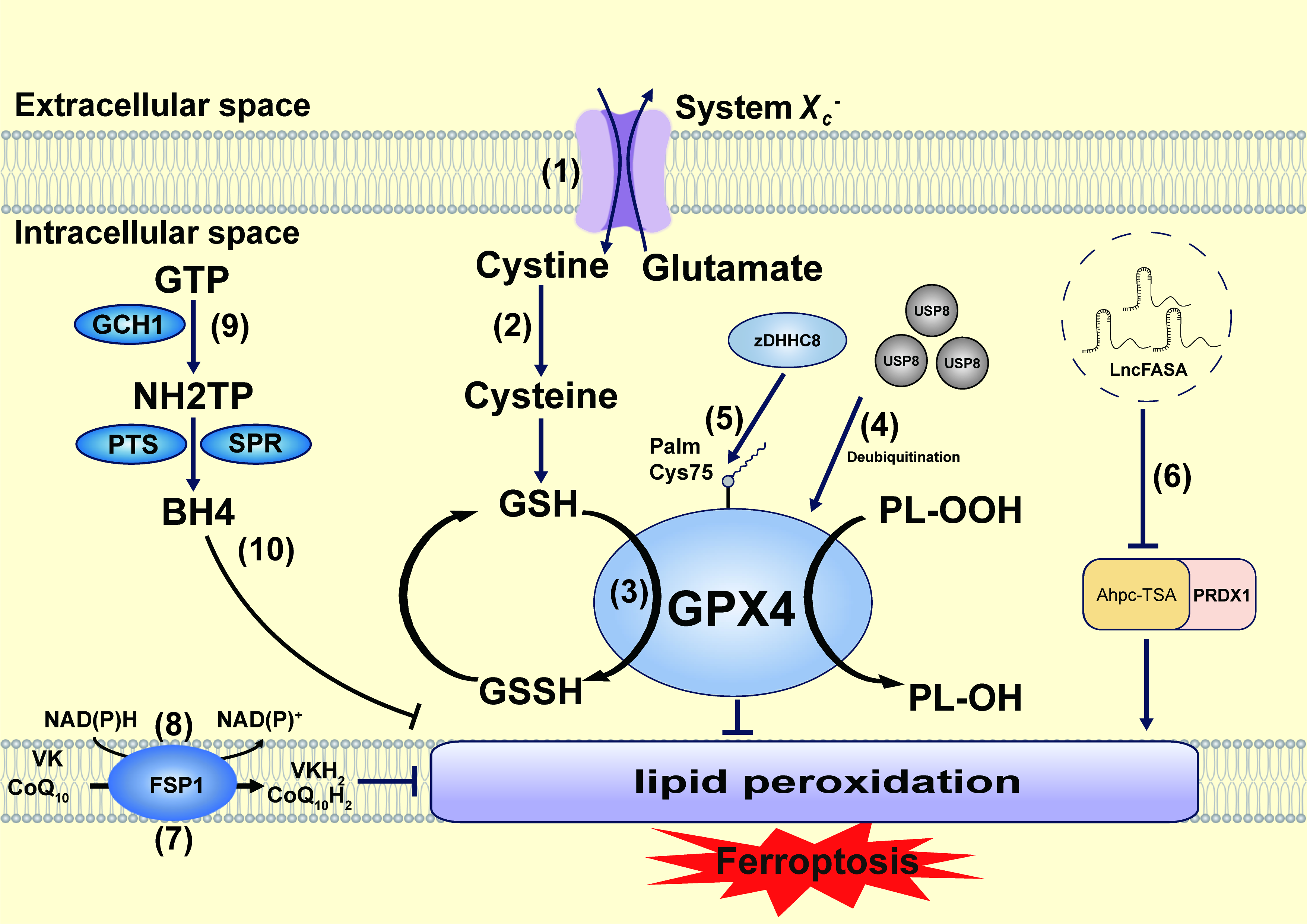

Supplement: Figure S1 [file OncolRes-34-69049-s001.tif]

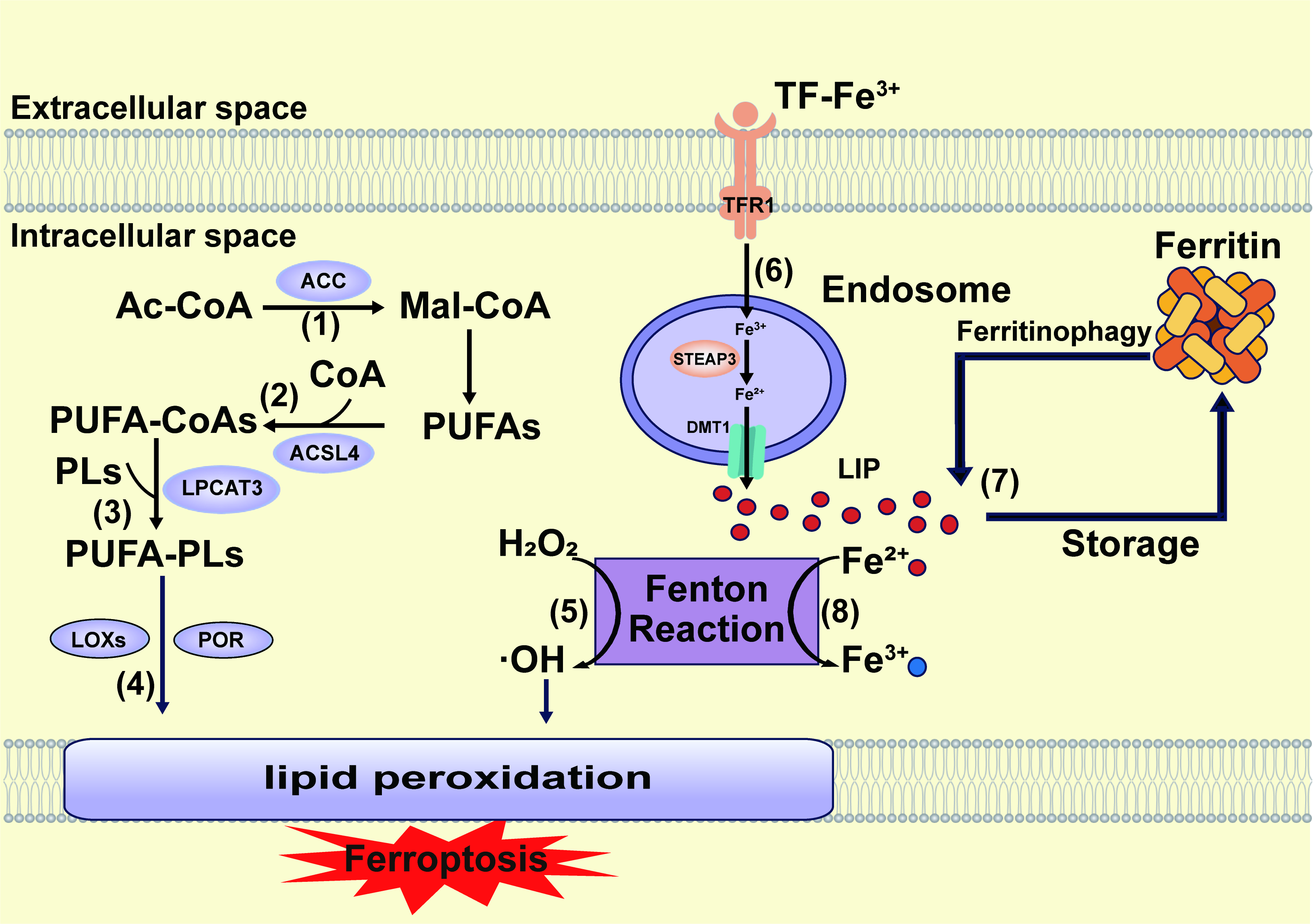

Supplement: Figure S2 [file OncolRes-34-69049-s002.tif]
