# Supplementary material for: Ferroptosis: Mechanisms, Comparison with Cuproptosis and Emerging Horizons in Therapeutics
Source: Oncol Res. 2025 Dec 30;34(1):8. doi: 10.32604/or.2025.069049 (PMC12774555; doi:10.32604/or.2025.069049)
Supplement: Supplementary file 3 [file OncolRes-34-69049-s003.docx]

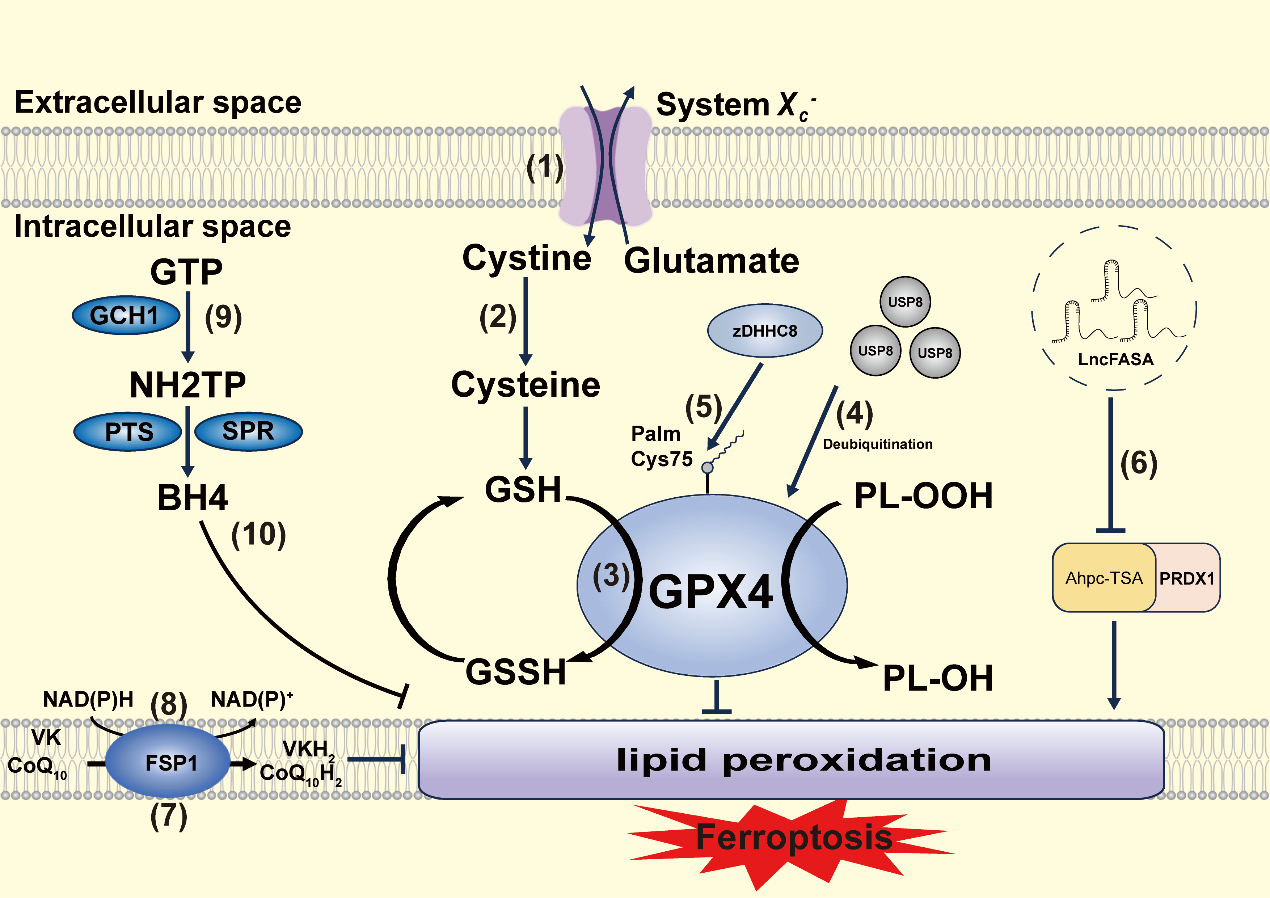


**Figure S1.** Concise schematic of ferroptosis defense mechanism. Some of the materials in the picture are taken from the drawing software "Biorender". (1) System Xc− imports cystine by exchanging intracellular glutamate (1:1). (2) Cystine is then reduced to cysteine. (3) GPX4 uses GSH to detoxify lipid hydroperoxides, suppressing lipid peroxidation. (4) USP8 stabilizes GPX4 through deubiquitination. (5) zDHHC8 mediates GPX4 palmitoylation at Cys75 to suppress ferroptosis. (6) PRDX1 suppresses ferroptosis by scavenging peroxides, whereas LncFASA inactivates PRDX1 through phase separation, leading to unchecked lipid peroxidation. (7) FSP1 generates ferroptosis-suppressive CoQ_10_H_2_ from CoQ_10_. (8) FSP1 reduces vitamin K to VKH2 using NAD(P)H, generating a radical-trapping antioxidant. (9) GCH1 converts GTP into NH_2_TP. (10) PTS and SPR work coordinately to produce BH4, which constitutes an enzymatic antioxidant defense system.


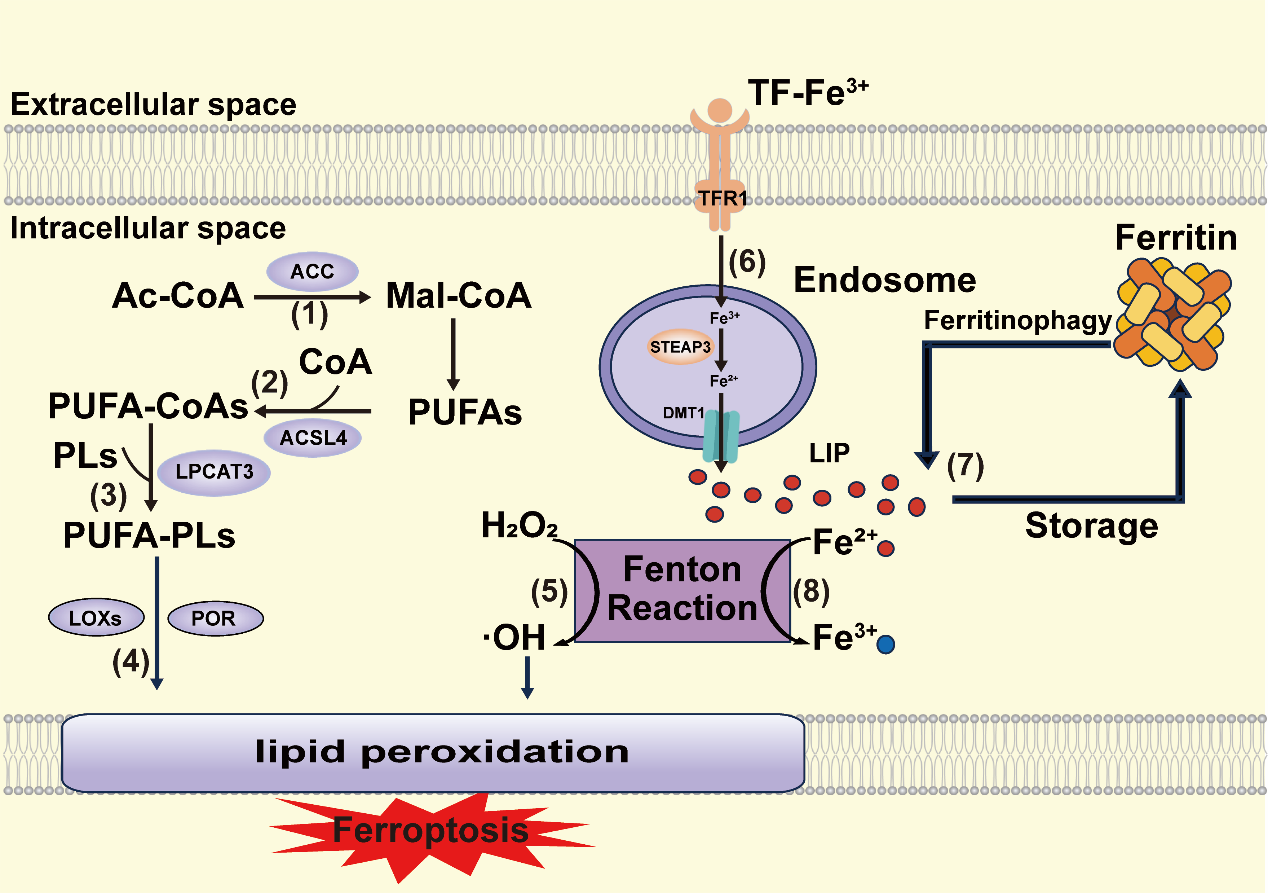
**Figure S2.** Simplified schematic diagram of the mechanism of ferroptosis. Some of the materials in the picture are taken from the drawing software "Biorender". (1) ACC catalyzes acetyl-CoA conversion to malonyl-CoA. (2) ACSL4 catalyzes the acylation of PUFAs with CoA, generating PUFA-CoAs. (3) LPCAT3 then incorporates PUFA-CoAs into PLs, forming PUFA-PLs. (4) LOXs and POR can initiate lipid peroxidation by catalyzing PUFA dioxygenation. (5) Free iron triggers Fenton reaction, generating excessive ROS. (6) TF-Fe³^+^ is taken up by TFR1, reduced to Fe²^+^ by STEAP3 in endosomes, and transported to the cytosol via DMT1, joining the LIP. (7) Ferritin sequesters iron but undergoes ferritinophagy, releasing stored iron into the LIP. (8) Excess Fe²^+^ in the LIP drives fenton reaction, triggering lipid peroxidation.
